# Study of gene function based on spatial co-expression in a high-resolution mouse brain atlas

**DOI:** 10.1186/1752-0509-1-19

**Published:** 2007-04-16

**Authors:** Zheng Liu, S Frank Yan, John R Walker, Theresa A Zwingman, Tao Jiang, Jing Li, Yingyao Zhou

**Affiliations:** 1 Department of Computer Science, University of California, Riverside, 900 University Avenue, Riverside, CA 92521, USA; 2 Genomics Institute of the Novartis Research Foundation, 10675 John Jay Hopkins Drive, San Diego, CA 92121, USA; 3 Allen Institute for Brain Science, 551 N 34th Street, Suite 200, Seattle, WA 98103, USA; 4 Electrical Engineering and Computer Science Department, Case Western Reserve University, 10900 Euclid Avenue, Cleveland, Ohio 44106, USA

## Abstract

**Background:**

The Allen Brain Atlas (ABA) project systematically profiles three-dimensional high-resolution gene expression in postnatal mouse brains for thousands of genes. By unveiling gene behaviors at both the cellular and molecular levels, ABA is becoming a unique and comprehensive neuroscience data source for decoding enigmatic biological processes in the brain. Given the unprecedented volume and complexity of the *in situ *hybridization image data, data mining in this area is extremely challenging. Currently, the ABA database mainly serves as an online reference for visual inspection of individual genes; the underlying rich information of this large data set is yet to be explored by novel computational tools. In this proof-of-concept study, we studied the hypothesis that genes sharing similar three-dimensional expression profiles in the mouse brain are likely to share similar biological functions.

**Results:**

In order to address the pattern comparison challenge when analyzing the ABA database, we developed a robust image filtering method, dubbed histogram-row-column (HRC) algorithm. We demonstrated how the HRC algorithm offers the sensitivity of identifying a manageable number of gene pairs based on automatic pattern searching from an original large brain image collection. This tool enables us to quickly identify genes of similar *in situ *hybridization patterns in a semi-automatic fashion and consequently allows us to discover several gene expression patterns with expression neighborhoods containing genes of similar functional categories.

**Conclusion:**

Given a query brain image, HRC is a fully automated algorithm that is able to quickly mine vast number of brain images and identify a manageable subset of genes that potentially shares similar spatial co-distribution patterns for further visual inspection. A three-dimensional *in situ *hybridization pattern, if statistically significant, could serve as a fingerprint of certain gene function. Databases such as ABA provide valuable data source for characterizing brain-related gene functions when armed with powerful image querying tools like HRC.

## Background

It is estimated that only ~1% of the genes expressed in human brain are studied in over 99% of the published neuroscience studies; we are far from understanding the enigmatic biological processes in the brain [1]. Microarray technology has been successfully applied to profile the expression landscape of the entire transcriptome in parallel; however, the size of typical brain samples dissected for mRNA extraction only allows the detection of a globally averaged expression level over a relatively large anatomical region; therefore, standard array based gene expression data sets often lack the desirable fine resolution required for neuroscience studies [2,3]. In order to preserve the relationships among brain circuitry, cell type, and gene expression, all of which are crucial for understanding the molecular machinery of the brain, *in situ *hybridization technology has been developed [4], which can be applied to measure the three dimensional high-resolution expression map of brain genes one at a time. The Allen Brain Atlas project [5,6], arguably one of the most ambitious post-genome projects, aims to systematically create a detailed gene expression brain atlas for as many as 24,000 genes by 2006. For each particular gene, 25 μm thick brain sections are cut at every 100–200 μm throughout the entire mouse brain. Hybridization of gene specific antisense probes to the brain slide enables quantitative measurement of the mRNA transcription level at an unprecedented cellular resolution. At the time of this study, data on 6080 genes were released online in the Allen Brain Atlas database [5]. At an estimated rate of generating 300 megabytes of map data per day [1], both the volume and the complexity of the image data present a difficult informatics challenge. Currently, the brain atlas database mainly serves as an online reference for visual examination of individual genes. The rich biological knowledge implied by this largest neuroscience database is yet to be explored–novel computational tools are essential for any such attempts.

Genes with similar expression profiles across a panel of different biological conditions are known to tend to share similar biological functions–a principle known as guilt by association (GBA) [7-11]. Extending the GBA concept to the brain atlas hypothesizes that genes share similar spatial brain expression landscapes could also imply similar biological functions. If validated, this idea will naturally become a powerful functional genomics tool for characterizing genes of unknown functions, as well as discovering new roles for known genes. One can envision a future version of the ABA database which provides an accurate pattern query and comparison tool to help neuroscientists discover genes of interesting spatial profiles and potential network partners in order to better understand the mechanism of a molecular target implicated in certain disease. In fact, ABA has made progress in this direction at the time of our writing.

To carry out such a proof-of-concept study, given a query gene of interest, we first have to develop an algorithm to help filter out obviously unrelated genes and highlight a manageable subset of genes that potentially share similar spatial expression patterns. Due to the complexity of the problem, the gene candidates discovered by the algorithm are then subjected to further human visual inspection, *i.e*., the sensitivity of the algorithm is more important given a reasonable specificity. In this study, we developed and compared three image similarity metrics required for gene filtering with increasing sophistication: a naïve pixel-wise metric, an adjusted pixel-wise metric, and a histogram-row-column (HRC) metric based on time series summary data. These three metrics were benchmarked and the superiority of the HRC algorithm was validated by cross validation studies. The biological studies presented in the Results and Discussion section are all made possible by using the HRC algorithm as a fully automated efficient first-pass filter.

We then studied several spatial hybridization patterns and showed that, in many cases, a selective brain atlas can represent an expression neighborhood that consists of genes of statistically enriched function categories. These discoveries were then cross validated using other related databases including the GNF Tissue Atlas [2], GenePaint.org [12], and the NCBI GENSAT database [13]. Most interestingly, our results illustrate how spatial co-expression leads to functional enrichment for the cyclic AMP (cAMP) regulatory pathway, particularly in relevance to adenylyl cyclase. We validated that substantia nigra enrichment serves as a signature pattern for the critical nigrostriatal dopaminergic pathway involved in Parkinson's disease after examining *Ddc*, *Slc6a3*, and *Slc18a2 *genes, which is consistent with the latest findings [14]. We conclude that the popular guilt by association principle can be aptly applied to the brain atlas database, transforming it into a rich source for functional genomic studies in neuroscience, in addition to a reference data repository.

## Results and discussion

### Measure the similarity of two brain images

The goal of this study is to investigate whether genes with similar spatial mRNA expression distribution in the brain tend to be functionally related. We first need to identify all the genes with similar mRNA expression to a given query gene based on the brain image at a particular slide location, and analyze the resultant gene list for any statistically significant functional enrichment based on existing biological annotations in the literature or gene ontology databases. Measuring the similarity of two brain images is a fairly complicated computational problem for several reasons. First, the ABA database, consisting of a growing large number of brain images, makes it nearly impossible for human manual inspection. At the time when this study began, the image data for 6080 genes were posted with dozens of images per gene corresponding to different brain anatomy locations. This number is increasing quite rapidly. For a single image query, over one million image pairs would need to be compared now. Second, in addition to the requirement of sophisticated data management solutions, the complexity of a brain image poses a significant computational challenge in terms of both image processing and pattern recognition. On top of these factors, brain samples are obtained from different mice, resulting in that the overall brain size and shape, as well as the contour of each brain anatomy region, can vary significantly even if one examines the same brain region at the same section position. Third, hybridization probes of different genes have heterogeneous biochemical properties, which could lead to varying hybridization signal intensity levels and potential cross-hybridization background levels across genes. One also needs to take into account the technical factors such as different sample orientations and image scanning artifacts in brightness and contrast. It is clear any algorithm that automatically measures the similarity of two brain images should be robust against the above mentioned biological and technical variations. However, due to the complexity of the problem, we do not expect such algorithm be good enough to replace human visual inspection, but should instead act as an automatic, efficient first-pass filter to highlight a subset of candidate gene slides, which is manageable for the second-pass visual refinement.

The gene expression level of an *in situ *hybridization is represented as an RGB image in the ABA database. The comparison of spatial expression between different images is actually an image registration problem, whose performance depends highly on the quality of the distance metric for an image pair. Typical image registration methods can either take the pixel intensity distribution or compute the pixel-by-pixel distances using Euclidean distance, Pearson correlation coefficient, etc. The most relevant approach to our study is the use of the Gaussian mixture model for expression distribution analysis [15]. But this method is not applicable in this case to analyze ABA images, because it lacks the capability of handling variations in anatomical regions across different brain slides. The parameters used in the global and local Gaussian mixture model matching do not reflect the gene expression property directly. Kumar *et al*. [16] uses the overlap between binarized images to measure distance. These metrics are designed to represent the global similarity or local similarity between images for different applications and are similar to our naïve pixel-wise algorithm. Here, we proposed three different alternative distance metrics for comparing a brain image pair with increasing complexity, namely naïve pixel-wise distance metric, adjusted pixel-wise distance metric, and a method based on intensity summaries by histogram, row, and column (so-called HRC method). It is noted that ABA has also released mask thumbnail images together with the original scans (mask images all have background, brightness, and contract factors corrected). Compared to the original hybridization scans, use of mask images has led to a significant performance improvement as expected. Also, at the end of our study, the ABA web site began to provide qualitative query features that enable a user to search genes based on "low/medium" or "high" expression levels in 11 selected brain regions. Carson et al. recently published a subdivision mesh technique for better pattern recognition of brain regions based on a set of reference slides; they provide web pattern query tools via GenePaint.org [14]. Compared to these recent developments, our method still offers the advantage of quantitative description of the expression patterns in an automatic fashion. We believe that both are important aspects for the future development of a large brain image database such as ABA.

### Training of the HRC weighting factors

A set of image pairs with "true" distances is required to train the weighting factors in the HRC method (see Methods) and to objectively benchmark the performance of various distance metrics. To construct such an unbiased data set, we resorted to the fact that slides of a given gene have similar texture patterns if they are obtained from close vicinities, while slides are most likely different if they are taken from brain regions far apart. Therefore, the physical distance between two slides of the same gene to some extent represents their "true" similarity.

A total of 1091 thumbnail sagittal slide images for 60 genes were downloaded from the ABA web site; the number of slides per gene ranges from 15 to 20. As described in Methods, the HRC weighting factor set that has the best average performance across all the 60 genes is chosen as the final, optimal weighting factors. We subsequently carried out a final run by combining all 60 genes as the training set and our final optimal weighting factors are [1.98, 107.39, 11.91] with an average Pearson correlation coefficient of 0.58.

### Comparison of the three distance metrics

**Figure 1:**
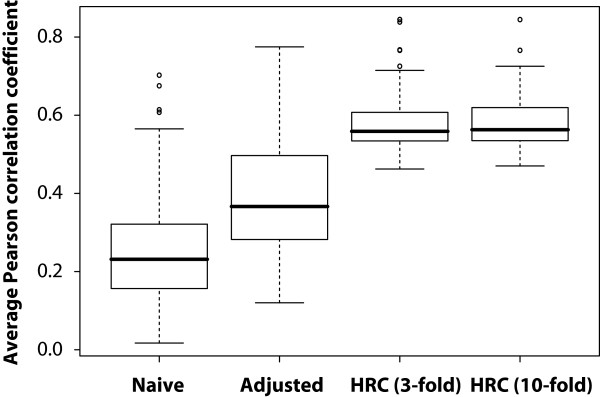
The performance of three algorithms benchmarked by a 60-gene test set. The performance improvement of the adjusted pixel-wise method versus the naïve pixel-wise method and the HRC algorithm versus the adjusted pixel-wise method are statistically significant (see also Table 1).

For each of the 60 genes used for testing, we first ran both the naïve pixel-wise algorithm and the adjusted pixel-wise algorithm on all the slide pairs within each genes and calculated a Pearson correlation coefficient between the resultant predicted slide distances *d*_*ab *_and their "true" distances dab∗
 MathType@MTEF@5@5@+=feaafiart1ev1aaatCvAUfKttLearuWrP9MDH5MBPbIqV92AaeXatLxBI9gBaebbnrfifHhDYfgasaacH8akY=wiFfYdH8Gipec8Eeeu0xXdbba9frFj0=OqFfea0dXdd9vqai=hGuQ8kuc9pgc9s8qqaq=dirpe0xb9q8qiLsFr0=vr0=vr0dc8meaabaqaciaacaGaaeqabaqabeGadaaakeaacqWGKbazdaqhaaWcbaGaemyyaeMaemOyaigabaGaey4fIOcaaaaa@31B1@. The methods show an average correlation coefficient of 0.27 and 0.40 for the 60 selected genes, respectively. Furthermore, in order to assess the performance of the HRC algorithm, we applied the standard *k*-fold cross validation method. The 60 genes were split into *k *partitions of equal size (for both *k *= 3 and *k *= 10 in this study), each combination of the *k*-1 partitions was in turn used as the training set to determine the optimal set of weighting factors and the remaining partition is used as the testing set to evaluate the performance of the HRC method (a total of 60 correlation coefficients, i.e. one per each gene). The procedure was then repeated 20 times to reduce statistical variance (a total of 1200 estimations); additional permutations did not lead to much change in the results. Specifically, in both the 3-fold and 10-fold cross-validation studies, the HRC algorithm achieved an average Pearson correlation coefficient of 0.58 (for both training set and testing set). Fig. 1 shows the box plot of the distribution of the correlation scores. Clearly, the HRC method outperforms both the naïve and adjusted pixel-wise algorithms.

In order to assess the performance of these three algorithms, statistical tests were applied to the obtained three groups of correlation coefficients. As shown in Table 1, both parametric two-tailed student *t*-test and non-parametric Wilcoxon test show that the enhancement of the adjusted method compared to the naïve one is highly statistically significant (*P*-value = 1.5 × 10^-5 ^and 1.8 × 10^-6^, respectively). This is in line with our expectation that the application of basic transformation operations can significantly improve the accuracy in recognizing similar brain images. Similar statistical tests show that the improvement of HRC with respect to the adjusted pixel-wise method is also statistically significant in both cases of 3- and 10-fold cross-validation simulations (*P *value < 10^-12^, Table 1). This indicates that the pixel-wise method is less robust against slide variations across different mouse samples, and by using a summary-based vector metric, the HRC algorithm indeed becomes more sensitive and can recognize slides in close vicinities.

### Application of the HRC method

Based on the above comparisons, the HRC algorithm is our final method of choice. Given the fact that the HRC algorithm is insensitive to the settings in the above cross validation tests, we are confident that given a particular gene slide of interest the HRC metric is able to help us filter out a large number of unrelated gene expression images without human intervention. We applied the HRC algorithm to study several genes of biological interest in order to assess the feasibility of carrying out a functional genomics study based on spatial gene expression in the mouse brain.

Since the 6080 ABA gene images were only available for online browsing, we manually downloaded 2759 sagittal brain slides of 145 genes for this proof-of-concept study. Given a gene of biological interest, we first identified a brain slide that shows interesting uncommon textual features and used it as our query image. We then applied the HRC algorithm to all the brain slides that are within 200 slide distances from the query slide position to rank these slides and locate genes with similar profiles. The HRC algorithm was applied recursively to the new set of genes that pass our visual inspection until a group of core genes with similar brain atlas expression patterns was obtained (based on visual judgment). Finally, we carried out biological functional analysis of the gene list based on literature search as well as other similar, smaller scale brain *in situ *hybridization databases. Several interesting examples have been found where the guilt by association principle can be applied to successfully establish the link between a characteristic gene spatial distribution pattern and a specific gene functional category. We summarized these findings in the following sections.

### Type 5 adenylyl cyclase is the primary isoform accountable for striatal adenylate cyclase activity

**Figure 2:**
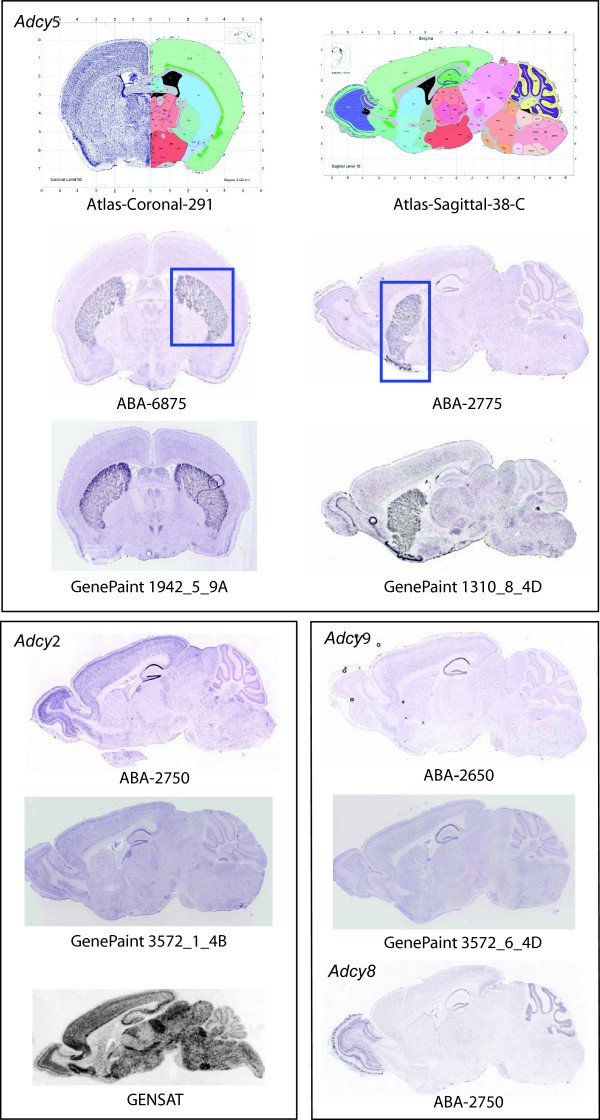
Spatial gene expression patterns of various adenylyl cyclase isoforms. *Adcy5 *is predominantly expressed in mouse striatum, while *Adcy2*, *Adcy8*, and *Adcy9 *show substantially different expression patterns. Brain images were obtained from the ABA, GenePaint.org, and GENSAT databases. The striatum region in ABA-6875 and ABA-2775 is outlined by a blue box.

The gene *Adcy5 *encodes adenylate cyclase 5, which is believed to be the major isoform responsible for the adenylyl cyclase activity in mouse striatum and was suggested to be a convergence site for both dopamine D_1 _and D_2 _signaling pathways [17,18]. Examination of both sagittal and coronal ABA images of this gene revealed that it is highly, if not exclusively, expressed in the striatum region of mouse brain, particularly caudoputamen (CP), nucleus accumbens (ACB), and olfactory tubercle (OT) (Fig. 2 and the two associated reference images Atlas-Sagittal-38-C and Atlas-Coronal-291). In addition, we examined other mouse adenylyl cyclase isoforms, such as *Adcy2*, *Adcy8*, and *Adcy9*, which are also selectively expressed in the brain. Available ABA images for *Adcy2*, *Adcy8*, and *Adcy9 *show no similarity in the spatial expression pattern compared to *Adcy5 *(Fig. 2), indicating the distinctive roles these isoforms may play in the mouse central nervous system. In fact, *Adcy5 *null mice express a dysfunctional motor phenotype consistent with a disruption in striatal dopamine signaling [17]. No general locomotor disruption was seen in *Adcy8 *null mice [19]. No such phenotype was described for *Adcy9 *null mice, and no information was available for *Adcy2 *null mice [20]. In this case, we were also able to cross-reference the corresponding *in situ *hybridization images from two other mouse brain databases: GenePaint [12] and NCBI GENSAT databases [13]. The images in these databases agree very well with the ABA data (Fig. 2). However, we did not find related data in the Mahoney Functional Genomic Atlas of the Mouse Brain database [21].

### Using a key component *Adcy5 *in the neuronal cyclic AMP signaling as a query pattern, the HRC algorithm is able to identify other proteins that are involved in this pathway

**Figure 3:**
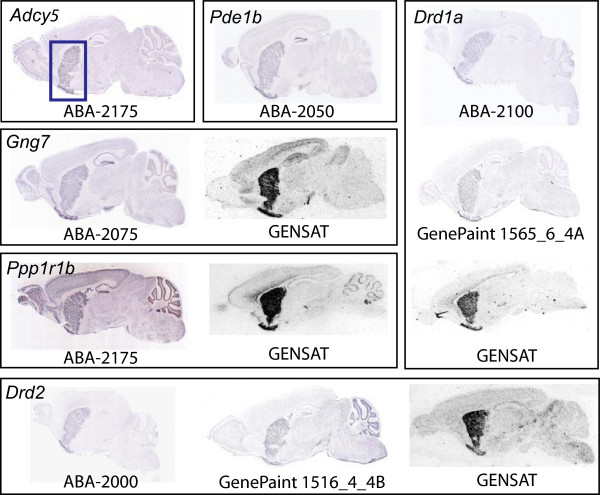
Genes involved in the neuronal cAMP signaling pathway share a similar striatum-enriched hybridization profile to *Adcy5*. Brain images were obtained from the ABA, GenePaint.org, and GENSAT databases. The striatum region is outlined in ABA-2175.

Given its striatum-specific expression and apparent involvement in the important nigrostriatal dopaminergic pathway [17,18], we applied *Adcy5 *as a query pattern (sagittal slide position 2175) to search the entire dataset obtained above using the HRC algorithm. We found that the top-ranked genes include *Pde1b*, *Gng7*, *Drd1a*, and *Drd2 *(Table 2 and Fig. 3). Specifically, *Pde1b *encodes Ca^2+^/calmodulin-dependent phosphodiesterase 1B, which is responsible for hydrolyzing cyclic nucleotide and therefore, presumably together with *Adcy5*, maintains cyclic AMP (cAMP) balance in mouse striatum. Moreover, *Drd1a *and *Drd2 *encode dopamine receptor D_1A _and D_2_, respectively, which are known to be involved in the neuronal cAMP signaling pathway [22]. Indeed, it has been demonstrated that diminished *Pde1b *activity increases cAMP signaling in response to dopamine D_1 _receptor agonist and consequently enhances dopaminergic function presumably via *Darpp32 *(also known as *Ppp1r1b*) and related pathways [23]. In addition, *Gng7 *encodes G protein γ_7 _subunit and a knockout study has shown that deletion of this gene results in diminished striatal adenylyl activity [24], consistent with its presumed involvement in the neuronal cAMP pathway. It seems that the guilt by association principle is also valid in terms of gene spatial distribution. Furthermore, as shown in Fig. 3 the ABA data also agree well with GenePaint and GENSAT images whenever available.

On one hand, we notice HRC algorithm indeed effectively identifies genes of relevant expression patterns. Among the top 20 genes in Table 2, five genes are known to be involved in adenylate cyclase activity and/or locomotory behavior based on an existing gene annotation database [25]. We further used Ingenuity Pathway Analysis (IPA) software [26] to study the related functions of these five genes, and it was found that they are all involved in the behavior function, mostly mouse locomotor activity, with a significance value of 10^-21^. Besides validating the HRC algorithm itself, the result also indicates that the *Adcy5 *expression pattern may be a signature pattern of the neuronal cAMP signaling pathway (Fig. 2). On the other hand, our visual inspection found that *Ppp1r1b*, which encodes protein phosphatase 1 regulatory subunit 1B, also shares similar expression pattern in the striatum region compared to *Adcy5*. The HRC algorithm was not able to identify it, despite the known fact that it is involved in the neuronal cyclic AMP signaling [22]. A closer examination of the ABA image revealed that *Ppp1r1b *is indeed highly expressed in the striatum region as *Adcy5*, while in the current ABA image it is also widely expressed in the cerebral cortex. This might prevent the HRC algorithm from high-ranking this gene. Nonetheless, based on the GENSAT image of *Ppp1r1b*, it is highly expressed mainly in the striatum region, bearing significant distribution similarity to *Adcy5 *(Fig. 3). *Gpr88 *is known to be a striatum specific G-protein coupled receptor [27], which also shares great sequence similarity with 5-HT_1D _receptor. Its strikingly similar spatial distribution with Adcy5 suggests that it might also be an uncharacterized gene involved in neuronal cAMP pathway. Knockout validations are being carried out.

### Cyclic AMP-regulated phosphoprotein 21 isoform 1 is the only gene product of *Arpp21 *involved in the striatal cAMP and Ca^2+^/calmodulin signaling pathway

**Figure 4:**
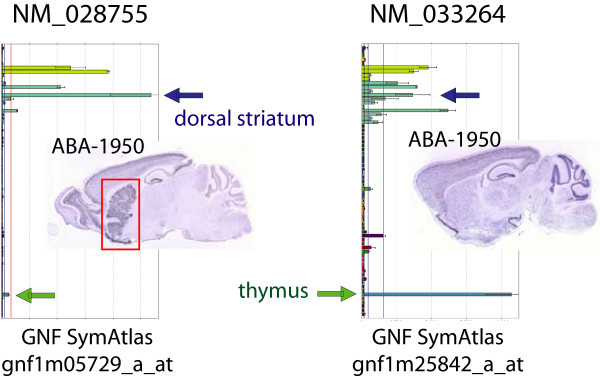
Spatial gene expression of the *Arpp21 *gene variants suggests only the isoform represented by RefSeq NM_028755 may be involved in the striatal cAMP signaling pathway. The striatum region is outlined by a red box. Brain images were obtained from the ABA database and the tissue gene expression data were obtained from GNF SymAtlas.

A previous study shows that gene *Arpp21 *(cyclic AMP-regulated phosphoprotein 21) encodes an important regulatory protein, regulator of calmodulin signaling (RCS), that is involved in the cellular cAMP signaling pathway regulated by protein kinase A (PKA) and protein phosphate 2B (PP2B), particularly in the striatal medium spiny neurons [22]. However, our initial search was not able to associate *Arpp21 *to the other members involved in the cAMP pathway, such as *Adcy5*, *Pde1b*, etc. We then used the UCSC Genome Browser to further examine the gene structure of *Arpp21*. It occurred to us that there are two transcript variants, namely RefSeq accession numbers NM_028755 and NM_033264, which encode *Arpp21 *isoforms 1 and 2, respectively. Based on the available data resources of GNF SymAtlas [28], which consists of gene expression data on 61 mouse tissues [2], we found two probe sets on the GNF1M Gene Chip which were designed from the above isoforms independently. Probe set gnf1m05729_a_at (NM_028755) shows high expression in dorsal straitum and low expression in thymus, while gnf1m25842_a_at (NM_033264) shows medium expression in dorsal straitum and high expression in thymus (Fig. 4). This suggested that the original *Arpp21 *probe designed by Allen Institute might be based on NM_033264 and a new probe using NM_028755 was desirable. Data on both variants of *Arpp21 *are now available from ABA after our initial proposal, a repeated searching using *Arpp21 *(RefSeq NM_028755) as the query gene on the new data collection found genes like *Adcy5*, *Gng7*, and *Pde1b *are among the top-ranked ones. As clearly shown in Fig. 3 and Fig. 4, *Arpp21 *isoform 1 (RefSeq NM_028755) is highly localized in the striatum region like others involved in the striatal cAMP pathway discussed above. The significant difference from the expression pattern of *Arpp21 *isoform 2 (RefSeq NM_033264), which initially appeared as a GBA outlier, is in fact due to splice variation.

### Key genes involved in the nigrostriatal dopaminergic pathway and Parkinson's disease are enriched in substantia nigra

**Figure 5:**
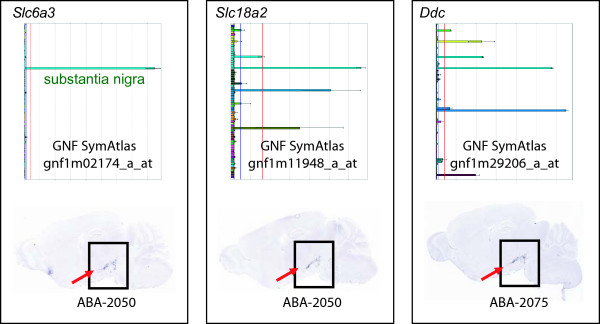
Gene expression of *Slc6a3*, *Slc18a2*, and *Ddc*. All three genes are enriched in the brain substantia nigra region as indicated by the arrow. Brain images were obtained from the ABA database and the tissue gene expression data were obtained from GNF SymAtlas.

The dopamine transporter (DAT), encoded by gene *Slc6a3 *[solute carrier family 6 (neurotransmitter transporter, dopamine), member 3], plays a critical role in the nigrostriatal dopaminergic pathway that is involved in the pathological development of Parkinson's disease [29,30]. The ABA images show that *Slc6a3 *expression is highly enriched in the substantia nigra (Fig. 5), in accordance with various previous studies [2,14,31]. We then applied *Slc6a3 *(slide position 2050) as the query pattern to search the dataset using the HRC algorithm. In the top 50 genes excluding *Slc6a3*, it contains *Lix1*, *Ptpru *(also known as *Ptprl*), *Lmx1b*, *Aldh1a1*, *Slc18a2*, and *Ddc*. This finding is consistent with a previous study that also employed mouse brain gene expression images [14]. In addition, three genes, namely *Aldh1a1*, *Ddc*, and *Slc18a2*, are found to be functionally annotated as "neurological disorder" by IPA with a significance value of 10^-4^. It is known that *Ddc*, *Slc18a2*, and *Slc6a3 *encode three major players in the dopaminergic nigrostriatal pathway, namely aromatic amino acid decarboxylase (AADC), vesicular monoamine transporter 2 (VMAT2), and dopamine transporter, respectively, and have been proposed to serve as biomarkers in the clinical evaluation of Parkinson's disease [29]. Also, the expression levels of these genes were found to decrease in animal models of Parkinson's disease [31].

It should be pointed out that *Slc6a3 *and related genes are expressed in a very small, localized region of the mouse brain (Fig. 5). This may create difficulty for the HRC method to carry out effective pattern matching for a small region, as information of all the rows and columns of the entire brain image is used to construct the row and column vectors, which may introduce noise into the ***H***, ***R***, ***C ***vectors. A potential improvement of this algorithm is to restrict the rows and columns used in creating the vectors based on specific region of interest of a query image. This may increase the sensitivity of the HRC method to discovery relevant matching brain images.

### Guilt by association on a three-dimensional level provides more information on gene function

**Figure 6:**
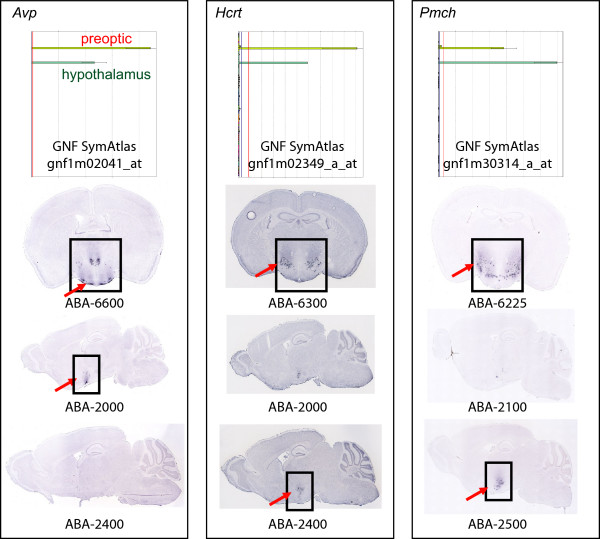
Gene expression of *Avp*, *Hcrt*, and *Pmch*. While little difference in expression among these genes is shown in the GNF mouse tissue atlas, the ABA images offer a better resolution and suggest *Avp *may play a different biological role than *Hcrt *and *Pmch*. The black boxes outline the brain hypothalamus region. *Avp *expression is concentrated in a specific hypothalamus region called periventricular region, while expressions of *Hcrt *and *Pmch *are largely concentrated in a different hypothalamus region called lateral hypothalamic area. Brain images were obtained from the ABA database and the tissue gene expression data were obtained from GNF SymAtlas.

According to the GNF mouse tissue atlas, we discovered that genes *Avp *(arginine vasopressin), *Pmch *(pro-melanin-concentrating hormone, also known as A230109K23Rik), and *Hcrt *(hypocretin) show nearly identical expression profiles in the hypothalamus and preoptic region of the hypothalamus dissections (Fig. 6). However, due to limitations in sample dissection, expression data obtained on a tissue level tend to measure a "smooth, average" expression level of a gene in a certain brain region. On the other hand, the expression data obtained from ABA images provide much greater detailed information on the three-dimensional distribution of a gene in mouse brain and hence enables us to study gene function with greater confidence based on the guilt by association principle. Indeed, as highlighted in Fig. 6 we were able to differentiate potential functions of *Pmch*, *Avp*, and *Hcrt *with greater resolution. Specifically, coronal slides in Fig. 6 show that *Hcrt *and *Pmch *are expressed quite broadly in the hypothalamus, distinctive from the expression pattern of *Avp*. On the other hand, *Avp *expression is concentrated in a specific hypothalamus region called periventricular region, in which neither *Hcrt *and *Pmch *are significantly expressed. Based on the guilt by association principle, it is likely that *Hcrt *and *Pmch *share related functions, which are different from that of *Avp*. This is in accordance with the available literature that *Hcrt*/*OX *(orexin) might have an effect on MCH (melanin-concentrating hormone) expression and they possibly interact coordinately [32]. In addition, the latest Brain Explorer from ABA is able to dynamically display the gene expression distribution on a three-dimensional level, which brings gene expression analysis to a new level and offers great assistance to the scientist.

### Future directions

At the current stage, we only tested hundreds of genes in this pilot study. There are certainly more research topics in exploring this unique ABA spatial gene expression data set. For example, after we filter out those dissemble images, it is very important to develop a more sophisticated method to rank the similar images in order to identify coregulated genes. Since similar images have high global similarity scores with the query image, we could focus on investigating the local similarity and spatial information to discover the most related images with confidence. In addition, we believe that quality control and sample standardization of mouse brain slides may greatly affect our ability in applying this image processing algorithm to the data, and hence special attention needs to be considered.

## Conclusion

We studied gene expression across the GNF and ABA atlases. With the help of our HRC filtering algorithm, we used the guilt by association approach to both confirm previous gene functional interactions and suggest new ones. Given query expression patterns of interest, we have shown that the HRC algorithm is able to produce a ranked gene list that is significantly enriched in visually confirmed positive hits and facilitates the discovery of signature patterns of important neurobiological pathways. We also highlighted the advantages of using this approach in databases of *in situ *hybridization images over microarray databases from tissue dissections. We believe a complete set of both coronal and sagittal mouse brain images will significantly facilitate confident (i.e. with statistical confidence) characterization of gene functions based on the unique information provided by ABA.

## Methods

### Image preprocessing

Brain images may have differential background intensities due to both biological and technical variations; therefore, the background effect should be removed before carrying out any meaningful comparison. As the background-subtracted mask images were made available by ABA during this study, they were used for all the calculations presented here and the background correction techniques will not be discussed. The mask brain slides outline the brain boundary, and pixels outside the brain region can be easily identified and their intensities were set to zero. The rest of the image has high contrast levels and can be closely approximated as binary black-and-white bitmap images. We first convert the mask slides into grayscale images and then into bitmap images based on the 128 intensity threshold. The resultant bitmap images led to better query results and were used for this study, although all the methods presented here are applicable to grayscale images as well.

### Naïve pixel-wise distance metric

Naive pixel-wise algorithm calculates a distance between two mask slides in a straightforward fashion. Given an image pair *a *and *b*, with **A **and **B **denoting their binary expression matrices, respectively. Only those pixels considered as foreground in both images (*F*_*a *_and *F*_*b*_) are taken into account, when comparing the two matrices. The naïve pixel-wise distance between *a *and *b*, denoted as *d*_*ab*_, is defined by the city-block/Manhattan distance as:

dab=∑i,j|Aij−Bij|∑i,j1, (i,j)∈Fa∩Fb
 MathType@MTEF@5@5@+=feaafiart1ev1aaatCvAUfKttLearuWrP9MDH5MBPbIqV92AaeXatLxBI9gBaebbnrfifHhDYfgasaacH8akY=wiFfYdH8Gipec8Eeeu0xXdbba9frFj0=OqFfea0dXdd9vqai=hGuQ8kuc9pgc9s8qqaq=dirpe0xb9q8qiLsFr0=vr0=vr0dc8meaabaqaciaacaGaaeqabaqabeGadaaakeaacqWGKbazdaWgaaWcbaGaemyyaeMaemOyaigabeaakiabg2da9maalaaabaWaaabuaeaadaabdaqaaiabhgeabnaaBaaaleaacqWGPbqAcqWGQbGAaeqaaOGaeyOeI0IaeCOqai0aaSbaaSqaaiabdMgaPjabdQgaQbqabaaakiaawEa7caGLiWoaaSqaaiabdMgaPjabcYcaSiabdQgaQbqab0GaeyyeIuoaaOqaamaaqafabaGaeGymaedaleaacqWGPbqAcqGGSaalcqWGQbGAaeqaniabggHiLdaaaOGaeiilaWIaeeiiaaYaaeWaaeaacqWGPbqAcqGGSaalcqWGQbGAaiaawIcacaGLPaaacqGHiiIZcqWGgbGrdaWgaaWcbaGaemyyaegabeaakiabgMIihlabdAeagnaaBaaaleaacqWGIbGyaeqaaaaa@5974@

where a pixel (*i*, *j*) is identified by its location at the *i*th row and the *j*th column of the image matrix.

### Adjusted pixel-wise distance metric

In addition to the basic background subtraction and contrast scaling that have been carried out for the mask slides, image pairs may require certain transformation operations such as translation and scaling in order to become more comparable. In the adjusted pixel-wise distance metric calculation, we address some of these factors, which may lead to an improvement in sensitivity. This method first linearly scales the height of foreground image *F*_*b *_to match the height of *F*_*a*_, then translates image *F*_*b *_horizontally with respect to *F*_*a *_in order to minimize *d*_*ab*_. We observed that for slides around the similar positions of the brain, their orientations are reasonably consistent. On the other hand, both sample size and shape differ significantly for slides with larger distance. Therefore, it is undesirable to perform the rotation optimization for such image pairs.

### Histogram-row-column (HRC) distance metric

**Figure 7:**
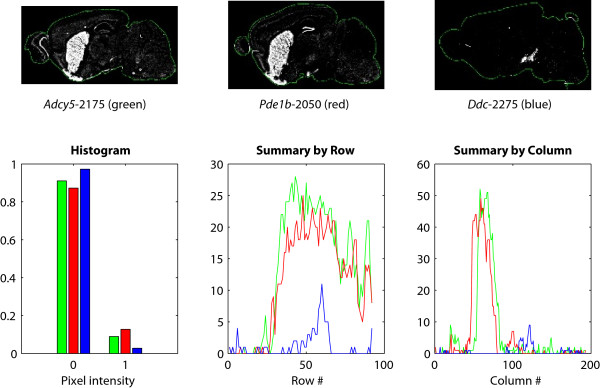
The mask images of slide 2175 of *Adcy5*, slide 2050 of *Pde1b*, and slide 2275 of *Ddc*. *Adcy5 *and *Pde1b *show similar expression profiles, while *Ddc *is expressed differently. The pixel intensity histogram, row vector, and column vector (***H***, ***R***, and ***C***, respectively) for the three genes are shown. Green represents *Adcy5*, red is for *Pde1b*, and blue is for *Ddc*.

The above two methods are both pixel-wise. Due to the complexity of the brain image, the adjustments carried out in the adjusted pixel-wise metric approach may not be sufficient and robust enough against various uncertain factors. Here we propose a non pixel-wise distance metric. The algorithm first performs all the steps in the adjusted pixel-wise method to minimize the distance between two mask slides according to Eqn. 1. We then generate three summary vectors to capture both global and local texture features of an image. Binary histogram ***H***, which simply counts the percentage of pixels ***H***_*k *_at each value *k *= 0 or 1, is a well known global summary metric. Sharing of a similar binary histogram is a necessary but not sufficient condition for two images to be considered similar. In order to address the drawback of omitting spatial distribution in the ***H ***vector, the binary matrix is further summarized into two additional vectors: a row vector ***R ***and a column vector ***C***. Specifically, ***R***_*i *_is calculated by summing the bits of all foreground pixels at row *i*; ***C***_*j *_is calculated by summing the bits of all foreground pixels at column *j*. ***H***, ***R***, and ***C ***can then be treated as time series data, where each time spot corresponds to a binary intensity, a row, or a column. Fig. 7 shows the ***H***, ***R***, and ***C ***vectors of three gene slides. It is clear that summary vectors between slide 2175 of *Adcy5 *and slide 2050 of *Pde1b *show a great deal of similarities, while those for slide 2275 of *Ddc *behave quite differently. This illustrates the basic idea of the HRC algorithm in sifting away brain slides that appear significantly different from the query slide. Distances between two images can be defined straightforwardly as:

dabH=∑k=0,1|Hka−Hkb|2
 MathType@MTEF@5@5@+=feaafiart1ev1aaatCvAUfKttLearuWrP9MDH5MBPbIqV92AaeXatLxBI9gBaebbnrfifHhDYfgasaacH8akY=wiFfYdH8Gipec8Eeeu0xXdbba9frFj0=OqFfea0dXdd9vqai=hGuQ8kuc9pgc9s8qqaq=dirpe0xb9q8qiLsFr0=vr0=vr0dc8meaabaqaciaacaGaaeqabaqabeGadaaakeaacqWGKbazdaqhaaWcbaGaemyyaeMaemOyaigabaGaemisaGeaaOGaeyypa0ZaaSaaaeaadaaeqbqaamaaemaabaGaemisaG0aa0baaSqaaiabdUgaRbqaaiabdggaHbaakiabgkHiTiabdIeainaaDaaaleaacqWGRbWAaeaacqWGIbGyaaaakiaawEa7caGLiWoaaSqaaiabdUgaRjabg2da9iabicdaWiabcYcaSiabigdaXaqab0GaeyyeIuoaaOqaaiabikdaYaaaaaa@4742@

dabR=∑i|Ria−Rib|∑iRia+∑iRib, i∈all rows
 MathType@MTEF@5@5@+=feaafiart1ev1aaatCvAUfKttLearuWrP9MDH5MBPbIqV92AaeXatLxBI9gBaebbnrfifHhDYfgasaacH8akY=wiFfYdH8Gipec8Eeeu0xXdbba9frFj0=OqFfea0dXdd9vqai=hGuQ8kuc9pgc9s8qqaq=dirpe0xb9q8qiLsFr0=vr0=vr0dc8meaabaqaciaacaGaaeqabaqabeGadaaakeaacqWGKbazdaqhaaWcbaGaemyyaeMaemOyaigabaGaemOuaifaaOGaeyypa0ZaaSaaaeaadaaeqbqaamaaemaabaGaemOuai1aa0baaSqaaiabdMgaPbqaaiabdggaHbaakiabgkHiTiabdkfasnaaDaaaleaacqWGPbqAaeaacqWGIbGyaaaakiaawEa7caGLiWoaaSqaaiabdMgaPbqab0GaeyyeIuoaaOqaamaaqafabaGaemOuai1aa0baaSqaaiabdMgaPbqaaiabdggaHbaaaeaacqWGPbqAaeqaniabggHiLdGccqGHRaWkdaaeqbqaaiabdkfasnaaDaaaleaacqWGPbqAaeaacqWGIbGyaaaabaGaemyAaKgabeqdcqGHris5aaaakiabcYcaSiabbccaGiabdMgaPjabgIGiolabbggaHjabbYgaSjabbYgaSjabbccaGiabbkhaYjabb+gaVjabbEha3jabbohaZbaa@61A0@

dabC=∑j|Cja−Cjb|∑jCja+∑jCjb, j∈all columns
 MathType@MTEF@5@5@+=feaafiart1ev1aaatCvAUfKttLearuWrP9MDH5MBPbIqV92AaeXatLxBI9gBaebbnrfifHhDYfgasaacH8akY=wiFfYdH8Gipec8Eeeu0xXdbba9frFj0=OqFfea0dXdd9vqai=hGuQ8kuc9pgc9s8qqaq=dirpe0xb9q8qiLsFr0=vr0=vr0dc8meaabaqaciaacaGaaeqabaqabeGadaaakeaacqWGKbazdaqhaaWcbaGaemyyaeMaemOyaigabaGaem4qameaaOGaeyypa0ZaaSaaaeaadaaeqbqaamaaemaabaGaem4qam0aa0baaSqaaiabdQgaQbqaaiabdggaHbaakiabgkHiTiabdoeadnaaDaaaleaacqWGQbGAaeaacqWGIbGyaaaakiaawEa7caGLiWoaaSqaaiabdQgaQbqab0GaeyyeIuoaaOqaamaaqafabaGaem4qam0aa0baaSqaaiabdQgaQbqaaiabdggaHbaaaeaacqWGQbGAaeqaniabggHiLdGccqGHRaWkdaaeqbqaaiabdoeadnaaDaaaleaacqWGQbGAaeaacqWGIbGyaaaabaGaemOAaOgabeqdcqGHris5aaaakiabcYcaSiabbccaGGqaciab=PgaQjabgIGiolabbggaHjabbYgaSjabbYgaSjabbccaGiabbogaJjabb+gaVjabbYgaSjabbwha1jabb2gaTjabb6gaUjabbohaZbaa@6522@

dab=wH⋅dabH+wR⋅dabR+wC⋅dabC
 MathType@MTEF@5@5@+=feaafiart1ev1aaatCvAUfKttLearuWrP9MDH5MBPbIqV92AaeXatLxBI9gBaebbnrfifHhDYfgasaacH8akY=wiFfYdH8Gipec8Eeeu0xXdbba9frFj0=OqFfea0dXdd9vqai=hGuQ8kuc9pgc9s8qqaq=dirpe0xb9q8qiLsFr0=vr0=vr0dc8meaabaqaciaacaGaaeqabaqabeGadaaakeaacqWGKbazdaWgaaWcbaGaemyyaeMaemOyaigabeaakiabg2da9iabdEha3naaBaaaleaacqWGibasaeqaaOGaeyyXICTaemizaq2aa0baaSqaaiabdggaHjabdkgaIbqaaiabdIeaibaakiabgUcaRiabdEha3naaBaaaleaacqWGsbGuaeqaaOGaeyyXICTaemizaq2aa0baaSqaaiabdggaHjabdkgaIbqaaiabdkfasbaakiabgUcaRiabdEha3naaBaaaleaacqWGdbWqaeqaaOGaeyyXICTaemizaq2aa0baaSqaaiabdggaHjabdkgaIbqaaiabdoeadbaaaaa@527A@

where *w*_*H*_, *w*_*R*_, and *w*_*C *_are three weighting factors that can be tuned based on their individual sensitivity in serving as independent distance metrics. Given a gene *g *of *n *slides, *n*^2 ^pair-wise image comparisons can be made. As mentioned above, when comparing two images the target image is transformed to optimally align with the query image; in this way, the algorithm might in principle perform slightly differently given different orders of two input images, and we count slide pair (*a*, *b*) and (*b*, *a*) as two different training instances. If *d*_*ab *_denotes the calculated distance according to Eqn. 5 between slide *a *and slide *b *of gene *g*, and dab∗
 MathType@MTEF@5@5@+=feaafiart1ev1aaatCvAUfKttLearuWrP9MDH5MBPbIqV92AaeXatLxBI9gBaebbnrfifHhDYfgasaacH8akY=wiFfYdH8Gipec8Eeeu0xXdbba9frFj0=OqFfea0dXdd9vqai=hGuQ8kuc9pgc9s8qqaq=dirpe0xb9q8qiLsFr0=vr0=vr0dc8meaabaqaciaacaGaaeqabaqabeGadaaakeaacqWGKbazdaqhaaWcbaGaemyyaeMaemOyaigabaGaey4fIOcaaaaa@31B1@ denotes the real physical distance between the two slides in the brain, the optimal set of weighting factors can then be determined by a global optimization routine:

{wHg,wRg,wCg}=arg⁡max⁡wH,wR,wCPearson(dab,dab∗|a,b∈all slide pairs for g).
 MathType@MTEF@5@5@+=feaafiart1ev1aaatCvAUfKttLearuWrP9MDH5MBPbIqV92AaeXatLxBI9gBaebbnrfifHhDYfgasaacH8akY=wiFfYdH8Gipec8Eeeu0xXdbba9frFj0=OqFfea0dXdd9vqai=hGuQ8kuc9pgc9s8qqaq=dirpe0xb9q8qiLsFr0=vr0=vr0dc8meaabaqaciaacaGaaeqabaqabeGadaaakeaadaGadaqaaiabdEha3naaDaaaleaacqWGibasaeaacqWGNbWzaaGccqGGSaalcqWG3bWDdaqhaaWcbaGaemOuaifabaGaem4zaCgaaOGaeiilaWIaem4DaC3aa0baaSqaaiabdoeadbqaaiabdEgaNbaaaOGaay5Eaiaaw2haaiabg2da9maaxababaGagiyyaeMaeiOCaiNaei4zaCMagiyBa0MaeiyyaeMaeiiEaGhaleaacqWG3bWDdaWgaaadbaGaemisaGeabeaaliabcYcaSiabdEha3naaBaaameaacqWGsbGuaeqaaSGaeiilaWIaem4DaC3aaSbaaWqaaiabdoeadbqabaaaleqaaOGaeeiuaaLaeeyzauMaeeyyaeMaeeOCaiNaee4CamNaee4Ba8MaeeOBa42aaeWaaeaacqWGKbazdaWgaaWcbaGaemyyaeMaemOyaigabeaakiabcYcaSiabdsgaKnaaDaaaleaacqWGHbqycqWGIbGyaeaacqGHxiIkaaGccqGG8baFcqWGHbqycqGGSaalcqWGIbGycqGHiiIZcqqGHbqycqqGSbaBcqqGSbaBcqqGGaaicqqGZbWCcqqGSbaBcqqGPbqAcqqGKbazcqqGLbqzcqqGGaaicqqGWbaCcqqGHbqycqqGPbqAcqqGYbGCcqqGZbWCcqqGGaaicqqGMbGzcqqGVbWBcqqGYbGCcqqGGaaicqWGNbWzaiaawIcacaGLPaaacqGGUaGlaaa@873B@

We then apply the weighting factors obtained from a particular gene *g *to the slides from all the other genes to assess their extrapolating performance. The factor set with the best average performance across the whole training set is then used.

## Authors' contributions

ZL developed the algorithm. SFY, JRW, TAZ, and YZ carried out most of the analyses. TJ and JL participated in the design of the study and helped algorithm development. ZL, SFY, and YZ drafted the manuscript. YZ conceived and coordinated the study. All authors have read and approved the final manuscript.

## Figures and Tables

**Table 1:** Comparisons of the three similarity metrics

**Comparison**	***P*-value (Student *t*-test)**	***P*-value (Wilcoxon test)**
Naïve vs. Adjusted	1.5 × 10^-5^	1.8 × 10^-6^
Adjusted vs. HRC (3-fold)	9.3 × 10^-13^	< 2.2 × 10^-16^
Adjusted vs. HRC (10-fold)	8.3 × 10^-13^	< 2.2 × 10^-16^

**Table 2:** Top-ranked genes identified using *Adcy5 *as the query pattern

**Similar gene**	**Distance**	**Slide position**	**Relevant GO term**
*Adcy5*	0	2175	cAMP biosynthesis; locomotory behavior
*Gpr88*	4.652	2100	G-protein coupled receptor protein signaling pathway
*Pde1b*	4.836	2050	locomotory behavior
*Gng7*	4.945	2075	locomotory behavior; receptor guanylyl cyclase signaling pathway
*Ppp1r2*	5.349	2150	
*Drd2*	6.938	2000	dopamine receptor, adenylate cyclase inhibiting pathway
*Drd1a*	7.123	2100	adenylate cyclase activation; locomotory behavior
*Dhcr24*	8.021	2150	cholesterol biosynthesis
*Sst*	8.193	2050	regulation of cell migration
*Fbxw11*	8.236	2325	
*Dhcr7*	8.260	2100	regulation of cell proliferation
*Lig1*	8.311	2000	DNA replication
*Calm3*	8.324	2150	G-protein coupled receptor protein signaling pathway
*Syt4*	8.331	2150	neurotransmitter secretion; regulation of calcium ion-dependent exocytosis
*Aldh1a1*	8.346	2350	embryonic eye morphogenesis
*Twsg1*	8.364	2100	
*Llglh*	8.365	2275	
*Igf2*	8.375	2000	organ morphogenesis
*No110_1700001E16Rik*	8.384	2025	
*Rrm1*	8.386	2225	
